# Supplementary material for: Family cohesion predicts long-term health and well-being after losing a parent to cancer as a teenager: A nationwide population-based study
Source: PLoS One. 2023 Apr 12;18(4):e0283327. doi: 10.1371/journal.pone.0283327 (PMC10096510; doi:10.1371/journal.pone.0283327)
Supplement: S1 Table — (DOCX) [file pone.0283327.s001.docx]

| **Supplementary Table 1.** | | |
| --- | --- | --- |
| **Questions** | **Responding options** | **Categorization** |
| Well-being |  |  |
| - Have you experienced high levels of well-being in the last month? | No, not at allYes, a littleYes, moderateYes, very high | Answering *No, not at all* or *Yes, a little* was categorized as low wellbeing |
| Quality of Life (QoL) |  |  |
| - Have you had a good quality of life the last month? | No, not at allYes, a littleYes, moderateYes, very good | Answering *No, not at all* or *Yes, a little* was categorized as low QoL |
| Depression |  |  |
| - PHQ-9 scale: Over the last 2 weeks, how often have you been bothered by any of the following problems?  1. Little interest or pleasure in doing things 2. Feeling down, depressed or hopeless 3. Trouble falling or staying asleep, or sleeping too much 4. Feeling tired or having little energy 5. Poor appetite or overeating 6. Feeling bad about yourself – or that you are a failure or have let yourself or your family down 7. Trouble concentrating on things, such as reading the newspaper or watching television 8. Moving or speaking so slowly that other people could have noticed? Or the opposite – being so fidgety or restless that you have been moving around a lot more than usual 9. Thoughts that you would be better off dead or of hurting yourself in some way | - Not at all (0 points) - Several days (about 1-3 days a week) (1 point) - More than half the days (about 4-5 days   a week) (2 points)   - Nearly every day (6-7 days a week) (points: 3) | Moderate to severe depression: total score ≥10 points |
|  | | |
|  | | |
| **Supplementary Table 1.** (Continued) | | |
| **Questions** | **Responding options** | **Categorization** |
| Symptoms of anxiety |  |  |
| - Have you felt persistent worries (fear, anxiety) in the last month? - Have you been unable to stop worrying or to control your worries in the last month? - Have you felt like something terrible is about to happen in the last month? - Have you had sudden attacks of anxiety (fear) in the last month? | - No - Yes, occasionally - Yes, approximately 1-3 days a week - Yes, approximately 4-5 days a week - Yes, approximately 6-7 days a week | Answering yes to once or more a week, to one or more of these questions, was considered indicating symptoms of anxiety. |
| Problematic sleeping |  |  |
| - Have you had trouble falling asleep at night in the last month? - Have you woken up during the night with anxiety or unpleasant feelings in the last month? | - No - Yes, occasionally - Yes, approximately 1-3 days a week - Yes, approximately 4-5 days a week - Yes approximately 6-7 days a week | Answering yes to once or more a week, to one or both of these questions, was considered indicating problematic sleeping. |
| Emotional numbness |  |  |
| - Have you felt emotionally numb (cut off, like you were in a bubble or had a wall around you) in the last month? | - No - Yes, occasionally - Yes, approximately 1-3 days a week - Yes, approximately 4-5 days a week - Yes, approximately 6-7 days a week | Answering yes to once or more a week, was used as an indicator for emotional numbness |
