# Supplementary material for: Family cohesion predicts long-term health and well-being after losing a parent to cancer as a teenager: A nationwide population-based study
Source: PLoS One. 2023 Apr 12;18(4):e0283327. doi: 10.1371/journal.pone.0283327 (PMC10096510; doi:10.1371/journal.pone.0283327)
Supplement: S2 Table — (DOCX) [file pone.0283327.s002.docx]

| **Supplementary Table 2.** | | | | | | |
| --- | --- | --- | --- | --- | --- | --- |
|  |  | **Family cohesion** | | | |  |
|  |  |  |  | |  |  |
| **Health-Related outcome, at time of the survey (6-9 years after the loss)** | **Total**  **n/total (%)** | **Poor ^a^**  **n/total (%)** | | **Good ^b^**  **n/total (%)** | | **Missing**  **n (%)** |
| **Low well-being the last month** | | | |  | |  |
| Whole group | 124/609 (20.4%) | 48/132 (36.4%) | | 76/477 (15.9%) | | 8 (1.3%) **^c^** |
| Paternally bereaved | 72/332 (21.7%) | 27/61 (44.3%) | | 45/271 (16.6%) | | 3 (0.5%) |
| Paternally bereaved males | 29/168 (17.3%) | 8/19 (42.1%) | | 21/149 (14,0%) | |  |
| Paternally bereaved females | 43/164 (26,2%) | 19/42 (45.2%) | | 24/122 (19,7%) | |  |
| Maternally bereaved | 52/276 (18.8%) | 21/71 (29.6%) | | 31/205 (15.1%) | | 5 (0.8%) |
| Maternally bereaved males | 16/134 (11.9%) | 8/20 (40%) | | 8/114 (7.0%) | |  |
| Maternally bereaved females | 36/142 (25.4%) | 13/51 (25.5%) | | 23/91 (25.3%) | |  |
| **Low quality of life the last month** | | | |  | |  |
| Whole group | 99/611 (16.2%) | 29/132 (22.0%) | | 70/479(14.6%) | | 6 (1.0%) **^c^** |
| Paternally bereaved | 52/332 (15.7%) | 13/61 (21.3%) | | 39/271 (14.4%) | | 3 (0.4%) |
| Paternally bereaved males | 25/168 (14.9%) | 6/19 (32.5%) | | 19/149 (12.8%) | |  |
| Paternally bereaved females | 27/164 (16.5%) | 7/42 (16.7%) | | 20/122 (16.4%) | |  |
| Maternally bereaved | 47/278 ((25.5%) | 16/71 (22.5%) | | 31/207 (14.4%) | | 3 (0.4%) |
| Maternally bereaved males | 16/134 (11.9%) | 5/20 (25.0%) | | 11/114 (9.6%) | |  |
| Maternally bereaved females | 31/144 (21.5%) | 11/51 (21.6%) | | 20/93 (21.5%) | |  |
| **Moderate/severe depression the last two weeks (PHQ-9)** | | | | | |  |
| Whole group | 83/610 (13.6%) | 42/133 (31.6%) | | 41/477 (8.6%) | | 7 (1.1%) **^c^** |
| Paternally bereaved | 42/329 (12.8%) | 19/62 (30.6%) | | 23/267 (8.6%) | | 6 (1.0%) |
| Paternally bereaved males | 17/167 (10.2%) | 7/19 (36.8%) | | 10/148 (6.8%) | |  |
| Paternally bereaved females | 25/162 (15.4%) | 12/43 (27.9%) | | 13/119 (10.9%) | |  |
| Maternally bereaved | 41/280 (14.6%) | 23/71 (32.4%) | | 18/209 (8.6%) | | 1 (0.2%) |
| Maternally bereaved males | 5/135 (3.7%) | 3/20 (15%) | | 2/115 (1.7%) | |  |
| Maternally bereaved females | 36/145 (24.8%) | 20/51 (39.2%) | | 16/94 (17.0%) | |  |

**^a^** Poor = no/little **^b^** Good = moderate/ very good **--** 1.00 [reference]

**^c^** The total number and % of missing values for each health-related outcome variable out of the 617 participant that answered the question regarding family cohesion (5 participants did not give an answer to that question or 0.8%). One participant did not state the gender of the deceased parent or the gender of the participant.

| **Supplementary Table 2.** (Continued). | | | | | | | |  |
| --- | --- | --- | --- | --- | --- | --- | --- | --- |
|  |  | **Family cohesion** | | | | |  | |
|  |  |  |  | |  |  |  | |
| **Health-Related outcome, at time of the survey (6-9 years after the loss):** | **Total**  **n/total (%)** | **Poor ^a^**  **n/total (%)** | | **Good ^b^**  **n/total (%)** | | | **Missing**  **n (%)** | |
| **Symptoms of anxiety once a week or more the last month** | | | | | | | |  |
| Whole group | 102/616 (16.6%) | 40/134 (29.9%) | | 62/482 (12.9%) | | | 1 (0.2%) **^c^** | |
| Paternally bereaved | 53/335 (15.8%) | 20/63 (31.7%) | | 33/272 (12.1%) | | | 0 (0.0%) | |
| Paternally bereaved males | 18/169 (10.7%) | 4/19 (21.1%) | | 14/150 (9.3%) | | |  | |
| Paternally bereaved females | 35/166 (21.1%) | 16/44 (36.4%) | | 19/122 (15.6%) | | |  | |
| Maternally bereaved | 49/280 (17.5%) | 20/71 (28.2%) | | 29/209 (13.9%) | | | 1 (0.2%) | |
| Maternally bereaved males | 14/134 (10.4%) | 3/20 (15.0%) | | 11/115 ((9.6%) | | |  | |
| Maternally bereaved females | 35/145 (24.1%) | 17/51 (33.3%) | | 18/94 (19.1%) | | |  | |
| **Problematic sleeping once a week or more the last month** | | | | | | | |  |
| Whole group | 123/616 (20.0%) | 41/134 (30.6%) | | 82/482 (17.0%) | | | 1 (0.2%) **^c^** | |
| Paternally bereaved | 71/335 (21.2%) | 24/63 (38.1%) | | 47/272 (17.3%) | | | 0 (0.0%) | |
| Paternally bereaved males | 30/169 (17.8%) | 9/19 (47.3%) | | 21/150 (14.0%) | | |  | |
| Paternally bereaved Females | 41/166 (24.7%) | 15/44 (34.1%) | | 26/122 (21.3%) | | |  | |
| Maternally bereaved | 52/280 (18.6%) | 17/71 (23.9%) | | 35/209 (16.7%) | | | 1 (0.2%) | |
| Maternally bereaved males | 19/135 (14.1%) | 2/20 (10%) | | 17/115 (14.8%) | | |  | |
| Maternally bereaved females | 33/145 (22.8%) | 15/51 (29.4%) | | 18/94 (19.1%) | | |  | |
| **Emotional numbness once a week or more the last month** | | | | | | | |  |
| Whole group | 77/610 (12.6%) | 34/132 (25.8%) | | 43/478 (9.0%) | | | 7 (1.1%) **^c^** | |
| Paternally bereaved | 38/332 (11.4%) | 16/61 (26.2%) | | 22/271 (8.1%) | | | 3 (0.5%) | |
| Paternally bereaved males | 15/168 (8.9%) | 4/19 (21.1%) | | 11/149 (7.4%) | | |  | |
| Paternally bereaved Females | 23/164 (14.0%) | 12/42 (28.6%) | | 11/122 (9.0%) | | |  | |
| Maternally bereaved | 39/277 (14.1%) | 18/71 (25.4%) | | 21/206 (10.2%) | | | 4 (0.6%) | |
| Maternally bereaved males | 15/134 (11.2%) | 6/20 (30.0%) | | 9/114 (7.9%) | | |  | |
| Maternally bereaved females | 24/143 (16.8%) | 12/51 (23.5%) | | 12/92 (13.0%) | | |  | |

**^a^** Poor = no/little **^b^** Good = moderate/ very good **--** 1.00 [reference]

**^c^** The total number and % of missing values for each health-related outcome variable out of the 617 participant that answered the question regarding family cohesion (5 participants did not give an answer to that question or 0.8%). One participant did not state the gender of the deceased parent or the gender of the participant.
