# Supplementary material for: Family cohesion predicts long-term health and well-being after losing a parent to cancer as a teenager: A nationwide population-based study
Source: PLoS One. 2023 Apr 12;18(4):e0283327. doi: 10.1371/journal.pone.0283327 (PMC10096510; doi:10.1371/journal.pone.0283327)
Supplement: Attachment — Submitted filename: Response to reviewers_2022.12.16.pdf [file pone.0283327.s003.pdf]

Response to reviewers

Editors comment #1
Please ensure that your manuscript meets PLOS ONE's style requirements, including those for file naming. The PLOS ONE style templates can be found at https://journals.plos.org/plosone/s/file?id=wjVg/PLOSOne_formatting_sample_main_body.pdf and https://journals.plos.org/plosone/s/file?id=ba62/PLOSOne_formatting_sample_title_authors_affiliations.pdf

- **Authors response to editors' comment #1**
  Dear editor we have now updated our documents in accordance to your style templates.

Editors comment #2
You indicated that you had ethical approval for your study. In your Methods section, please ensure you have also stated whether you obtained consent from parents or guardians of the minors included in the study or whether the research ethics committee or IRB specifically waived the need for their consent.

- **Authors response to editors' comment #2**
  Dear editor, all of the participants where adults at the time of participation (18 to 26 years of age), therefore no consent was needed from their parents who no longer were considered to be their guardians at the time of the study according to Swedish laws.

Editors comment #3
Thank you for stating the following in the Acknowledgments Section of your manuscript:
We would also like to thank the Swedish Cancer Foundation [2008–758], The Gålö Foundation, The Kamprad Family Foundation for Entrepreneurship, Research and Charity, and The Mats Paulssons Stiftelse for supporting the research project with grants.

However, funding information should not appear in the Acknowledgments section or other areas of your manuscript. We will only publish funding information present in the Funding Statement section of the online submission form.
Please remove any funding-related text from the manuscript and let us know how you would like to update your Funding Statement. Currently, your Funding Statement reads as follows:

The Swedish Cancer Foundation (2008–758), https://www.cancerfonden.se (GS);
The Kamprad Family Foundation for
Entrepreneurship, https://familjenkampradsstiftelse.se/in-english/ (CJ); The Mats Paulsson Foundation, https://www.matspaulssonstiftelserna.com (CJ), and the Gålö
Foundation, https://www.galostiftelsen.se (UK) supported the research project. The funders had no role in study design, data collection and analysis, decision to publish, or preparation of the manuscript.
Please include your amended statements within your cover letter; we will change the online submission form on your behalf.

- **Authors response to editors' comment #3**
  Dear editor we have now removed all funding information from the section additional information in the manuscript (**line 865** in the *Revised Manuscript with tracked changes*). The information in the funding statement is correct and not in need of an update and therefore no amended statements regarding this are included in the cover letter.

Editors comment #4
We note that you have indicated that data from this study are available upon request. PLOS only allows data to be available upon request if there are legal or ethical restrictions on sharing data publicly. For more information on unacceptable data access restrictions, please see http://journals.plos.org/plosone/s/data-availability#loc-unacceptable-data-access-restrictions.

In your revised cover letter, please address the following prompts:

a) If there are ethical or legal restrictions on sharing a de-identified data set, please explain them in detail (e.g., data contain potentially sensitive information, data are owned by a third-party organization, etc.) and who has imposed them (e.g., an ethics committee). Please also provide contact information for a data access committee, ethics committee, or other institutional body to which data requests may be sent.

We will update your Data Availability statement on your behalf to reflect the information you provide.

- **Authors response to editors' comment #4**
  Dear editor we have now removed the data availability statement from the Additional Information section of the manuscript (see **line 865** in the *Revised Manuscript with tracked changes*). To make our Data availability statement clearer, please make the suggested update to the data availability statement of the manuscript, remove the text that is marked with strikethrough and add the text that is shown in red below:

  In order to assure data confidentiality and to protect the privacy of the research participants, the datasets generated and/or analyzed during the current study are not publicly available due to legal and ethical restrictions as described by the Swedish law (2003:460) and the Swedish Ethical Review Authority (https://etikprovningsmyndigheten.se) regarding processing of sensitive data ~~of sensitive nature, but are~~. Data can be made available from the Department of Oncology-Pathology, Division of Clinical Cancer Epidemiology, Karolinska Institute, Stockholm, Sweden *(contact via* gunnar.steineck@ki.se) *~~corresponding authors on reasonable request~~, for researchers who meet the criteria for access to confidential data.*

Editors comment #5
Your ethics statement should only appear in the Methods section of your manuscript. If your ethics statement is written in any section besides the Methods, please delete it from any other section.

- **Authors response to editors' comment #5**
  We have now removed the ethical statement that was listed under the section Additional information in the manuscript. (See **line 865**) and moved it to the Methods section (see **lines 144–150**)

Editors comment #6
Please include captions for your Supporting Information files at the end of your manuscript, and update any in-text citations to match accordingly. Please see our Supporting Information guidelines for more information: http://journals.plos.org/plosone/s/supporting-information.

- **Authors response to editors' comment #6**
  We have now added a Supporting information heading and caption for our Supporting information files in **lines 773–777** and we have updated the in-text citations, see **lines 197** and **482** in the *Revised Manuscript with tracked changes*.

Editors comment #7
Please review your reference list to ensure that it is complete and correct. If you have cited papers that have been retracted, please include the rationale for doing so in the manuscript text, or remove these references and replace them with relevant current references. Any changes to the reference list should be mentioned in the rebuttal letter that accompanies your revised manuscript. If you need to cite a retracted article, indicate the article's retracted status in the References list and also include a citation and full reference for the retraction notice.

- **Authors response to editors' comment #7**
  Thank you we have now reviewed our reference list and made the following changes:
  - **Reference nr. 1** has been updated and now cites the publisher's final edited version of the article.
  - **Reference nr. 21** appeared two times in the reference list (ref. nr. 21 and 39) in the previous submission of the manuscript. The duplicate has now been removed.
  - **References nr 24** and **25** are new to the reference list, cited in **line 105** and **106** in the revised manuscript.
  - **Reference 29**, available from link has now been added to the reference.
  - **Reference nr. 33** is new to the reference list, cited in **line 135** in the revised manuscript.
  - **Reference nr. 58** is new to the reference list, cited in **line 546** in the revised manuscript.
  - **Reference 67**, available from link has now been added to the reference.
  - **Available DOI, PMID and PMCID have been added** to the references in the reference list, when applicable.

<u>Reviewer #1 - Comments</u>

The manuscript "Family cohesion predicts long-term health and well-being after losing a parent to cancer as a teenager: A nationwide population-based study" is very well written, the results are very well presented, the statistical analysis is well done, and it will potentially promote preventive interventions in bereaved teenagers.

However, the concept of family has been changing during the last decades. IMHO the authors started from a traditional family concept without considering different families, socio-economical and educational levels, cultural and religious backgrounds etc.
In addition, the composition of families was not described at all. The concept of living together when the parent dies is an administrative requirement, but what about brothers and sisters, grandparents, etc.? There are no specifications.

Consequently, the definition of family cohesion should be described to understand what we are discussing as a potentially protected factor.
I highly recommend considering these questions face to future generations of family members. I suggest briefly defining the "family" you managed and why. Furthermore, what do you consider "family cohesion" to mean? You also should include some discussion about these concepts and your results.

- **Authors response to reviewers' comments**

  Dear reviewer we would like to thank you for taking the time to review our manuscript and for your useful comments to our manuscript.

  <u>Regarding family cohesion:</u>
  The definition of family cohesion that was given in the background section of the manuscript has now been updated (see **lines 110–111** in the *Revised Manuscript with tracked changes*).

  The aim of the study was to measure the participants own subjective experience of family cohesion and was measured with a single item question as described in the method section. This means that we cannot know what the phenomenon of family cohesion means to each participant since we have only measured their own perception of the level of family cohesion. We have now clarified this in **lines 538–545** in the *Revised Manuscript with tracked changes*.

  We have also suggested further research to probe deeper into the concept of family cohesion in **lines 548–552** and under clinical implications in **lines 595–598** in the *Revised Manuscript with tracked changes*.

  <u>Regarding the family construction:</u>
  Regarding the reviewers' quest for better defining the family, we have now added in the Introduction section a text that describes the concept of family based on Bowens family systems theory (see **lines 103–106** in the *Revised Manuscript with tracked changes*).

  Since this was a nationwide population-based study, all those who had lost a parent to cancer during their teenage years in the reference years and who had been living in a

two parents' household were invited to participate, this included people from all geographical areas within the country of Sweden and of different socioeconomic statuses. Additionally, the participants also needed to be born in one of the Nordic countries and speak and understand Swedish.

Factors such as income, education and occupational status are often used to indicate the socioeconomic status. We did not collect any data on the participants or their parents' income level, but in table 2 the educational level of the parents and the participants current employment status, are stated, which can give an indication of the socioeconomic status. An information about the number of siblings of the participants is also shown in table 1, but information about other social circumstances, such as support from grandparents or religious background was not within the scope of this paper.

To address why first-generation immigrants' and other forms of family constellations than two-parents' households were excluded from the study, has now been clarified in the methodological discussion, **lines 504–513** in the *Revised Manuscript with tracked changes*.

<u>Reviewer #2: - Comments</u>

Reviewer #2: Parentally bereaved children are an underserved population. This study is important as it adds information to help healthcare professionals understand the need for family support after the death of a parent and highlights the long-term impact of parental loss during adolescence.
The only question I have concerns using the cutoff of 16 versus 18 years old for age at the time of parental death. Why not include adolescents who had lost a parent between the ages of 13-18 years old?

- **Authors response to reviewers' comments**
  Dear reviewer we would like to thank you for your comments to our manuscript. We have now clarified the rationale behind restricting the age of the participants to 13-16 years of age at the time of death (see **lines 132–135** in the *Revised Manuscript with tracked changes*).
